# Controlled synthesis of Cu nanoparticle arrays with surface enhanced Raman scattering effect performance†

**DOI:** 10.1039/c7ra10694g

**Published:** 2018-01-08

**Authors:** Qianqian Ding, Lifeng Hang, Liang Ma

**Affiliations:** Department of Precision Manufacturing Engineering, Suzhou Vocational Institute of Industrial Technology Suzhou 215104 China; Department of Polymer Science and Engineering, School of Chemistry and Materials Science, University of Science and Technology of China Hefei 230026 P. R. China maliangs@mail.ustc.edu.cn

## Abstract

Herein, we report the synthesis of a 350 nm Cu nanoparticle array with different period combinations by a method based on a monolayer and have further investigated its surface-enhanced Raman scattering (SERS) properties experimentally. The SERS properties of the 350 nm Cu nanoparticle array were investigated, and the influence of the thickness of the Cu nanoshell was studied. The results demonstrated that the 18 min ion-sputtering deposition can improve the SERS activity in addition to good stability. This study can provide an optimized method for some inexpensive nanomaterials as highly active SERS substrates and a good solution to the interference caused by substrate impurity.

## Abstract 2

Herein, we report the synthesis of a 350 nm Cu nanoparticle array with different period combinations by a method based on a monolayer and have further investigated its surface-enhanced Raman scattering (SERS) properties experimentally.

## Graphical abstract

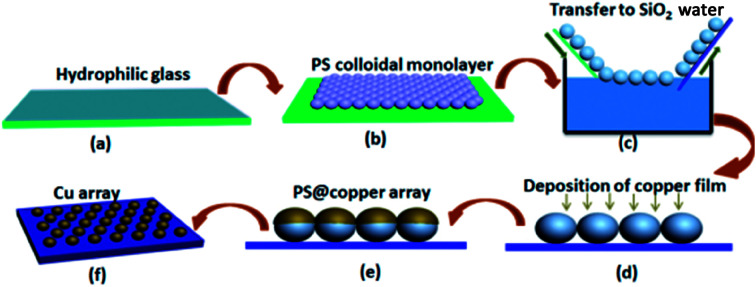

## 1. Introduction

SERS has attracted significant attention as it is a highly sensitive analytical technique, and it has already been widely employed in a variety of fields including analytical chemistry, environmental monitoring, medical science, and explosives analysis.^1–3^ As is known, the SERS phenomenon is observed primarily from the surface of Au, Ag, Cu, and other noble metals.^4,5^ It is generally believed that Ag-based substrates may provide highest electromagnetic enhancement leading to a large enhancement of Raman intensity, but they have intrinsically low chemical stability towards surface oxidation, which greatly hinders their application in various fields.^6^ In contrast, Au-based SERS substrates have long-term stability although they can provide moderate enhancement in SERS experiments.^7^ However, the Au and Ag materials are too expensive to come into practice. Therefore, development of new substrates with low price and better stability is of great significance. Among all the metals, Cu nanomaterials have the similar localized surface plasmon resonance (LSPR) effect as the Au and Ag nanomaterials.^8–10^ Moreover, Cu nanomaterials are rich in the earth's crust and inexpensive. Therefore, Cu is expected to replace these noble metals in many different areas such as in catalysis, surface-enhanced Raman scattering, and so on.^11–13^

At present, Cu nanoparticles are normally prepared by the reduction of a Cu precursor and solution condition. Cu nanoparticles are normally prepared through Cu ion reduction. This method needs surfactants for stability. It is still difficult to achieve large area mono-dispersion when chemical reduction method is used to prepare Cu nanoparticles.^14,15^ Other methods to prepare nanoparticles are grinding or thermally evaporating the solid Cu.^16^ The disadvantage of this method is the difficulty to control the morphology of the nanoparticles. Therefore, the preparation method of a Cu array with an ordered structure is still very challenging.

**Fig. 1:**
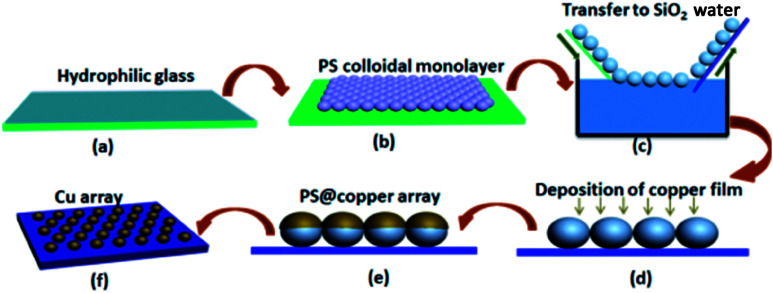
Schematic of the preparation of the Cu nanosphere array. (a) The hydrophilic glass substrate with a layer of water; (b) a PS monolayer colloidal crystal was fabricated on a cleaned glass slide by an air/water interfacial self-assembly method; (c) PS microsphere array was transferred onto the SiO_2_ substrate through liquid surface transfer; (d–e) microsphere array after ion-sputtering deposition of the Cu film on the surface of the PS nanosphere array; (f) the PS colloidal array with Cu coating was annealed under a protective atmosphere, and the Cu nanosphere array was formed on the SiO_2_ substrate.

In this research, polystyrene spheres (PS) were used as a template, and the outer shell of Cu was obtained based on the PS monolayer colloidal crystal template *via* the ion-sputtering deposition method (strategy shown in Fig. 1). The SERS activity of these Cu nanoparticles was evaluated using crystal violet (CV) and 4-aminothiophenol (4-ATP) as the probe molecules. The experimental result shown was that the 18 min ion-sputtering deposition could improve the SERS activity in addition to good stability. This study provides an effective way to prepare an inexpensive Cu SERS substrate, which can provide an optimized method for using Cu as a highly active SERS substrate.

## 2. Experimental

### 2.1 Preparation of the Cu nanosphere array

The Cu nanosphere arrays were prepared through the ion-sputtering method, and the synthetic process is shown in Fig. 1. At first, the 350 nm uniform monolayer colloidal PS crystal was synthesized on a piece of well cleaned glass slide by the gas–liquid–solid interface self-assembly method, as illustrated in previous research.^17,18^ After drying at room temperature, a copper layer was successively deposited on it using the ion-sputtering deposition method. After this, we optimized the sputtering time, and a series of microsphere arrays with different particle sizes was obtained.

### 2.2 Characterization

The morphology of the Cu microsphere array was characterized by FE-SEM (S-4700, Hitachi, Japan) and X-ray energy dispersive spectroscopy (EDS) at an acceleration voltage of 200 kV. Transmission electron microscopy (TEM) images were obtained by the JEOL 2010 high resolution transmission electron microscope operating at an acceleration voltage of 200 kV. Raman spectra were obtained using a confocal microscope Raman system (LabRAM HR800, Horiba Jobin Yvon, Japan) at 532 nm. The radiation generated by an air-argon ion laser (Spectra-Physics model 163-C4260) was the excitation source.

## 3. Results and discussion

### 3.1 Cu microsphere array

**Fig. 2:**
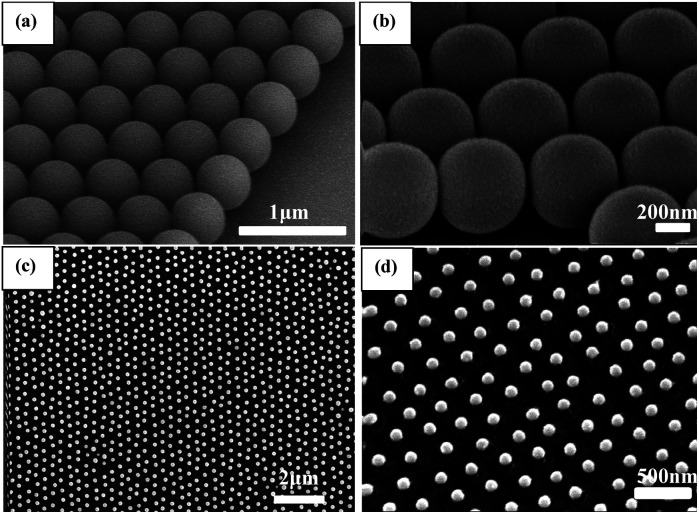
(a) SEM images observed from the tilted view of the 350 nm PS monolayer colloidal crystal and PS@Cu composite microsphere (b); (c) SEM images of Cu nanoparticles with a periodic length of 350 nm observed from the top view; and (d) SEM images of Cu nanoparticles with a periodic length of 350 nm observed from the tilted view.

Fig. 1 shows the synthetic process of the Cu nanosphere array. The uniform 350 nm polystyrene sphere (PS) monolayer colloidal crystal was fabricated on a piece of well cleaned glass slide by the self-assembly method of air/water interface. Fig. S1† displays the SEM images of the 350 nm PS colloidal monolayer on the glass slide, which shows the hexagonal close-packed alignment. The PS@Cu composite nanosphere array was thus obtained *via* successively ion-sputtering deposition of Cu on the PS colloidal monolayer with different thicknesses. Upon comparing the SEM images of the PS nanosphere and PS@Cu composite microsphere from the tilted view, we found that the PS nanosphere was smooth, and the surface of the PS@Cu microsphere was rough. The round nanosphere changed to elliptic morphology, as shown in Fig. 2b. The PS@Cu composite microsphere array was calcined under a protective atmosphere at 900 °C for 2 h. During the annealing process, each PS sphere was removed from the array, and most of Cu coated on it when the surface of the sphere was melted and formed a spherical nanoparticle. This was driven by the *in situ* principle of surface free energy minimum. The different stages about the annealing processes of the copper nanospheres are shown in Fig. S2.† When the temperature was increased, the copper film shrank to form spherical Cu NPs *in situ* from a spherical shell to spherical nanoparticles. Hence, the Cu nanosphere array was developed on the SiO_2_ substrate. Fig. 2c and d show the SEM images of Cu nanoparticles with a periodic length of 350 nm observed from the top and tilted view, respectively. We observed the Cu nanoparticles with a size of *ca.* 110 nm with the hexagonal non-closed-packed (hncp) arrangement. The whole Cu nanosphere array maintained a good order on the SiO_2_ substrate. Moreover, energy dispersive spectrometry (EDS) of the Cu nanoparticles indicates that the sample only contains two elements: Cu and Si, as shown in the inset images of Fig. S3.†

**Fig. 3:**
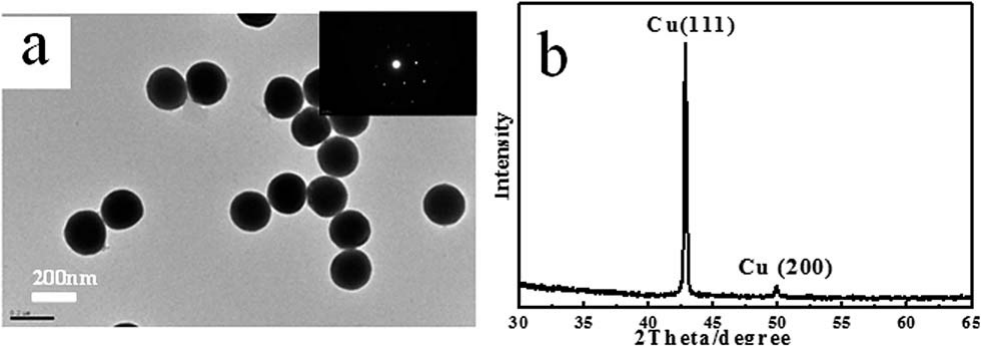
(a) TEM images of Cu nanoparticles and (b) XRD patterns of the Cu nanoparticles.

TEM images in Fig. 3 show a typical surface morphology of Cu nanoparticles. From the TEM image, we have found that the Cu nanoparticles have an obvious spherical shape and uniform size, and the surface is very smooth without any impurities. The selected area electronic diffraction (SAED) is used to accomplish the micro morphology observation and electron diffraction. The diffraction pattern shown in Fig. 3a has very symmetrical diffraction spots, which indicates that nanoparticles are single crystals. Further analysis of the particles shows that the single crystal has face-centred-cubic (FCC) structures. The samples were also characterized by X-ray diffraction (XRD). The identified Cu peaks were assigned to the face-centred cubic structures, and the peaks at 42.8° and 49.9° were attributed to (111) and (200), respectively.

**Fig. 4:**
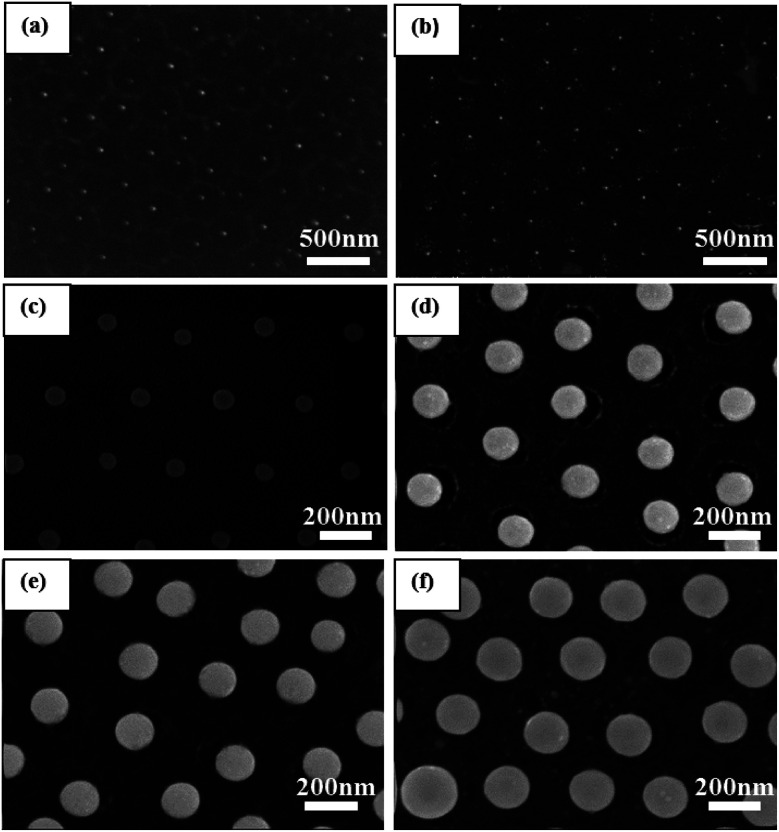
SEM images of the Cu nanoparticles with different deposition times: (a) 1 min, (b) 2 min, (c) 3 min, (d) 6 min, (e) 9 min, and (f) 18 min, the deposition current is 20 mA.

We adjusted the deposition time to control the size of the Cu nanoparticles. Controlling the thickness of the deposition plays a key role in forming an orderly nanoparticles array. When the thickness of the deposited Cu film is too low or too high, the Cu nanoparticle array cannot form. The 350 nm PS monolayer colloidal crystal was used as the template. The time of ion-sputtering deposition is different, and the thickness of Cu shell is different. Fig. 4 shows the SEM images of Cu nanoparticles annealed from different predecessors. When the deposition time was 1 min and the current was 20 mA, the PS nanospheres disappeared after annealing, and the uneven Cu nanoparticles had a small size in the hexagon area. As shown in Fig. 4a, in the area of each hexagon profile, there is a particle, but the size of the particle is very uneven and has no profile. It suggests that the 1 min deposition time is too short to form a neat and orderly array. As shown in Fig. 4b, when the deposition time is 2 min, the large Cu nanoparticle distribution with multiple small particles is in the area of each hexagon profile. When the deposition time is 3 min, the Cu nanoparticles are significantly bigger than before, and the surrounding small particles disappear. The diameter of Cu nanoparticles is about 70 nm (shown in Fig. 4c). When the deposition time increased to 6 min and 9 min, the diameter of the Cu nanoparticles is 120 nm (shown in Fig. 4d) and 150 nm, respectively (shown in Fig. 4e), and the morphology of the Cu nanoparticles is uniformly spherical and show a hexagonal non-closed-packed arrangement. When the deposition time was increased to 18 min, Cu nanoparticles became uneven (shown in Fig. 4f). To sum up, for the 350 nm PS monolayer colloidal crystal as the template, the deposition time should be 3–18 min, and the diameter will be 70–200 nm (the deposition current was 20 mA).

### 3.2 Cu nanoparticle array for SERS substrates

**Fig. 5:**
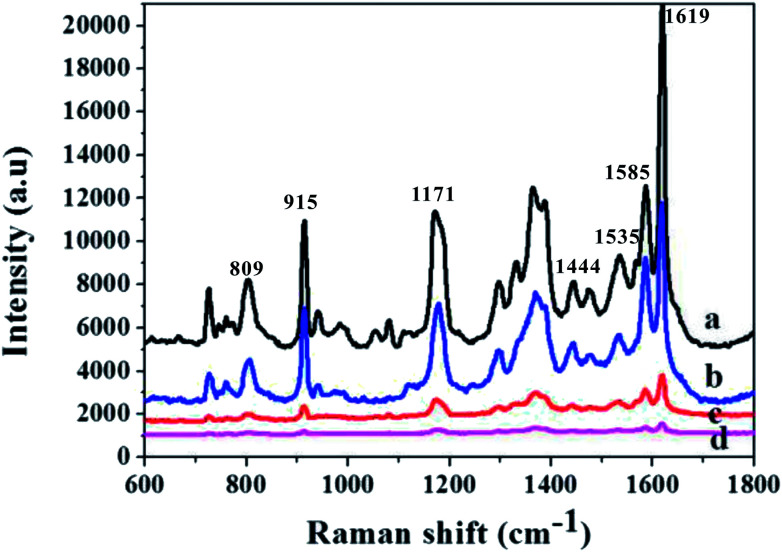
Surface enhanced Raman scattering spectra of 10^−6^ M CV on the Cu nanoparticle arrays with different deposition times.

Cu nanomaterials have a similar LSPR effect as Au and Ag nanoparticles. Thus, the Cu nanoparticle array can be used for the SERS substrate. Because of the periodic structure, the SERS signals are stable with high reproducibility. The Cu nanoparticle arrays with deposition time were first immersed in 10^−6^ M crystal violet (CV) solution for 30 min and subsequently rinsed with deionized water several times to remove the free or physically adsorbed molecules. After drying under ambient conditions in air, SERS characterization was conducted. Fig. 5 shows the SERS signal intensities of CV absorbed on the Cu nanoparticle arrays with different diameters. The CV molecules exhibit vibrational bands corresponding to the aromatic C–C stretching modes at 1619, 1585, 1535, and 1444 cm^−1^, an N-phenyl stretching mode at 1370 cm^−1^, and aromatic C–H bending modes at 1171, 915, and 809 cm^−1^. As can be seen, the 3 min deposition time has the weakest SERS signals (cave d). The Cu nanoparticles with 18 min deposition time produce the relatively significantly enhanced SERS signal (cave a). The SERS substrate at 18 min deposition time has a significant enhancement due to the inclusion of more particles in the unit area under 18 min deposition time. In addition, since the spacing between the particles is in the tens or even hundreds of nanometers, the coupling effect is not considered. Really playing a role of coupling effect is the superposition of the entire individual granules SERS effect light panel. The results demonstrate that the 18 min ion-sputtering deposition can improve the SERS activity. To observe the enhancement intuitively and quantitatively of the 18 min ion-sputtering deposited Cu nanoparticle arrays, the SERS enhancement factor (EF) was calculated as follows:EF = (*I*_SERS_/*N*_SERS_)/(*I*_0_/*N*_0_)*I*_SERS_ and *I*_0_ are the peak intensities of the SERS measurement and the regular Raman measurement, respectively. *N*_SERS_ and *N*_0_ represent the numbers of the corresponding surface and solid molecules effectively excited by the laser beam, respectively.^19^ We used the 10^−10^ M concentration for an estimate of the ensemble EF. On the basis of the intensity of the aromatic C–C stretching modes at 1619 cm^−1^ (Fig. S4†), the average EF was calculated to be 1.6 × 10^7^.

**Fig. 6:**
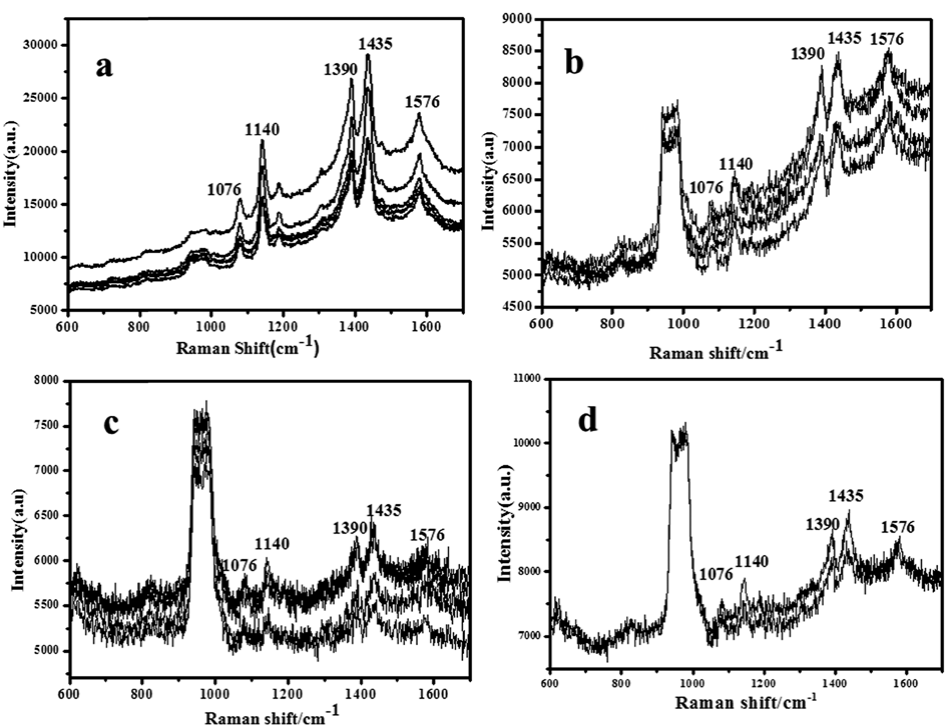
Surface enhanced Raman scattering spectra for the Cu nanoparticle arrays probed with the various concentrations of 4-ATP, 10^−4^ M (a), 10^−5^ M (b), 10^−6^ M (c), and 10^−7^ M (d).

The sensitive properties of the Cu nanoparticle arrays will be demonstrated through SERS detections. 4-ATP solution with different concentrations (10^−4^, 10^−5^, 10^−6^, and 10^−7^ M) was prepared. Then, 2 μL 4-aminothiophenol (4-ATP) was dropped on 0.3 × 0.3 cm^2^ Cu nanoparticle arrays with 18 min ion-sputtering deposition. In each sample, 4–5 points are selected, and the test results are shown in Fig. 6. Herein, two sets of bands were observed in the SERS spectra of 4-ATP on the surface of Cu NPs: one set is located at 1076 and 1576 cm^−1^, which is assigned to the a1 vibrational modes, and the other set is located at 1140, 1390, and 1435 cm^−1^, which is assigned to the b2 vibrational modes. The Cu nanoparticle arrays with 18 min ion-sputtering deposition has a good SERS enhancement effect on 4-ATP of 10^−4^ M. The SERS signal of the selected 5 points is stable, and the width peak at the position 900 cm^−1^ is from the base SiO_2_ (shown in Fig. S5†). When the 4-ATP concentration was reduced, the SERS signals gradually decayed, and the concentrations of 10^−5^ and 10^−6^ M used also showed very good SERS enhancement effect until 10^−7^ M. Integral time was increased to 14 s to measure the effect of the weaker concentration. No signal was detected beyond 10^−7^ M; thus, no experimental results were obtained for it.

## 4. Conclusions

In summary, the SERS activity of simple Cu nanoparticle arrays, which were fabricated by a one-step ion-sputtering deposition method on a PS colloidal monolayer template, with different periodic lengths was investigated using CV as the probe molecule. The experimental results demonstrate that the 18 min ion-sputtering deposition can improve the SERS activity, and using the calcination process, the surfactant interference with the SERS effect is reduced. The SERS substrates have good stability and reproducibility. All these properties used for the SERS substrates open up a new opportunity for inexpensive SERS measurement.

## Conflicts of interest

There are no conflicts of interest to declare.

## Supplementary Material

RA-008-C7RA10694G-s001
