# Supplementary material for: Controlled synthesis of Cu nanoparticle arrays with surface enhanced Raman scattering effect performance
Source: RSC Adv. 2018 Jan 8;8(4):1753–7. doi: 10.1039/c7ra10694g (PMC9077090; doi:10.1039/c7ra10694g)
Supplement: RA-008-C7RA10694G-s001 [file RA-008-C7RA10694G-s001.pdf]

Electronic Supplementary Material (ESI) for RSC Advances.
This journal is © The Royal Society of Chemistry 2017

Supporting Information

Controlled synthesis of Cu nanoparticles arrays with surface enhanced Raman scattering effect performance

Qianqian Ding‡,[a] Lifeng Hang‡[b] and Liang Ma*[b]

Experimental Section

Chemicals: Monodispersed polystyrene (PS) microsphere suspensions (2.5 wt% in water, surfactant-free) with 350nm sizes were purchased from Alfa Aesar Corporation. The target of copper (purity, 99.99%; 60*4 mm) was bought from ZhongNuo Advanced Material (Beijing) Technology Corporation. The Si wafers were used as substrates for self-assembling of PS spheres. Mixed gas (N_2 (98%), H_2 (2%)) was bought from Nanjing Special Gas Factory Corporation. Graphite powder, acetone, ethanol, H_2O_2, $NH_3.H_2O$, $NaBH_4$ and 4-Aminothiophenol (4-ATP) were of analytical grade and were bought from Sinopharm Chemical Reagent Co. Ltd. Deionized water used in all experiments (18.2 M U cm) was obtained from ultrafiltration system.

Fabrication of periodic Cu nanosphere arrays:

The SiO_2 wafers were washed with ethanol, acetone and deionized water in the ultrasonic bath in sequence, then cleaned in $H_2O/H_2O_2/NH_3.H_2O$ (3:1:1 in volume) and deionized water in turn, followed by drying in the oven at 90 ^{0}C for 20 min. After above cleaning steps, the substrates were sufficiently hydrophilic. Subsequently, PS microsphere suspensions were mixed evenly with the same volume of ethanol by the ultrasonic bath for 15 min. Finally, a large-area colloidal monolayer was fabricated on the cleaned substrate by interfacial self-assembling process. Colloidal monolayer templates with 350nm periodic length on the SiO_2 wafers were placed on the platform of sputtering device (K550X) for copper deposition. During sputtering process, the sputtering current of 20 mA was applied and the deposition process was performed with different time from 3 to 28 min according to colloidal templates with the periodicities from 350 nm to 750 nm. The colloidal monolayer templates after copper deposition were annealed at 900 C for 2 h under the mixed gas (N_2 (98%), H_2 (2%)).

TEM sample preparation: The annealed Cu nanospheres on the SiO_2 wafers were immersed into the ethanol. Then the sample was treated with ultrasonic apparatus for 30 min. The obtained solution was dropped onto the copper grid.

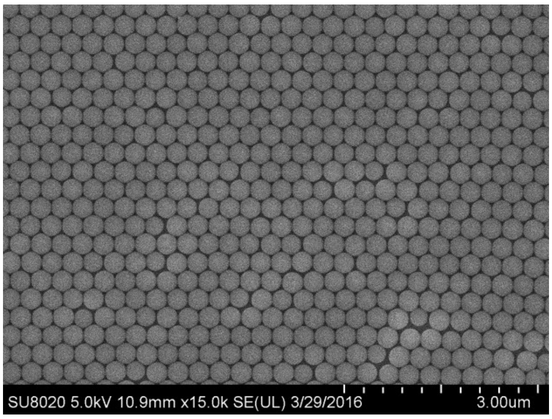

Fig.S1 the SEM images of the 350nm PS colloidal monolayer.

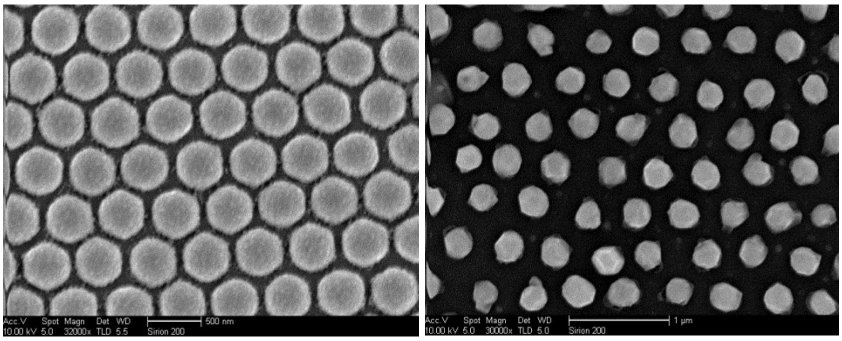

Fig.S2 SEM images of copper nanoparticle with different stages

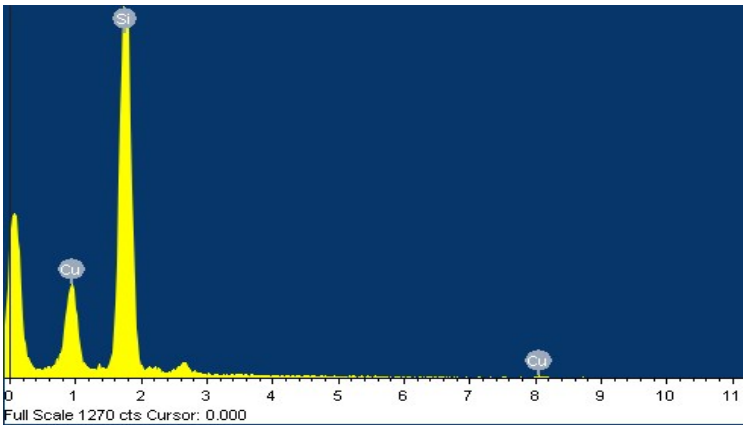

Fig.S3 EDS spectrum of the Cu nanoparticles

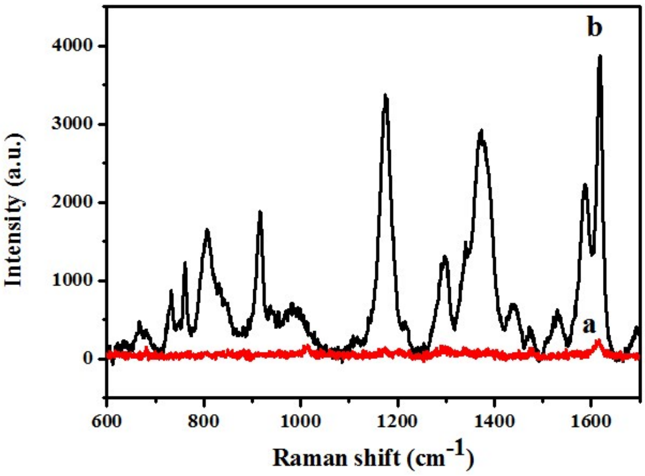

Fig. S4 Raman spectrum of 0.1M CV (curve a) and SERS spectra of 10^{-10}M CV (curve b) obtain from Cu nanoparticles arrays with the 18 min ion-sputtering deposition.

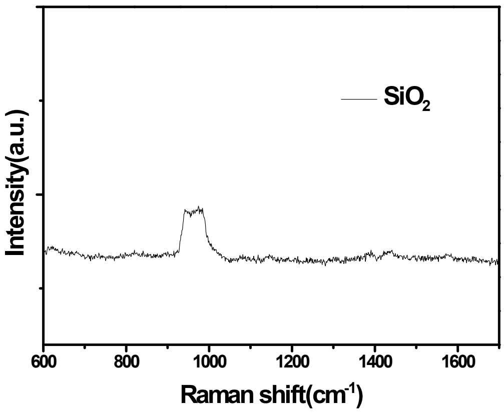

Fig. S5. The Raman spectrum of SiO_2 wafers.
